# First report of IS*Kpn26* element mediating *mgrB* gene disruption in the ST1 colistin- and carbapenem-resistant *Klebsiella pneumoniae* cluster isolated from a patient with chest infection

**DOI:** 10.1128/spectrum.00952-24

**Published:** 2024-09-24

**Authors:** Xiaosi Li, Siquan Shen, Yan Feng, Heping Shen, Fupin Hu, Xiaoyan Wu

**Affiliations:** 1 Department of Laboratory Medicine, The Second Affiliated Hospital of Jiaxing University, Jiaxing, China; 2 Institute of Antibiotics, Huashan Hospital, Fudan University, Shanghai, China; 3 Department of Laboratory Medicine, Jiaxing Maternity and Child Health Care Hospital, College of Medicine, Jiaxing University, Jiaxing, China; 4 Department of Infectious Disease, The Second Affiliated Hospital of Jiaxing University, Jiaxing, China; Duke University, Durham, North Carolina, USA

**Keywords:** carbapenem-resistant *Klebsiella pneumoniae*, *bla*_KPC-2_, colistin, *mgrB*, ST1

## Abstract

Colistin is used as a last-line therapy against carbapenem-resistant *Klebsiella pneumoniae* (CRKP). However, colistin resistance in *Klebsiella pneumoniae* is increasingly reported worldwide. This study aims to investigate the instrumental role of insertion sequence (IS) elements in colistin resistance through *mgrB* disruption in *K. pneumoniae* during treatment. Five clinical isolates of CRKP, designated KPN1~KPN5 were collected from the lower respiratory tract of a patient with chest infection before and after treatment with colistin. Antimicrobial susceptibility testing was performed using the broth microdilution method. Whole genome sequencing and bioinformatics were used to analyze the sequence types (STs), resistance genes, and genetic characteristics of the five isolates of *K. pneumoniae*. Antimicrobial susceptibility testing indicated that all five *K. pneumoniae* isolates were resistant to cephalosporins (ceftriaxone, ceftazidime, and cefepime), several carbapenems (imipenem, meropenem), cefoperazone–sulbactam, piperacillin–tazobactam, ciprofloxacin, and fosfomycin, whereas they were sensitive to amikacin and tigecycline. In addition, three of these isolates were resistant to colistin, with minimum inhibitory concentration values of >8 mg/L. Whole genome sequencing revealed that all five *K. pneumoniae* isolates belonged to sequence type 1 (ST1), which shared an identical *bla*_KPC-2_. Notably, disruption of *mgrB* by the IS*Kpn26* insertion sequence was shown to be the primary colistin resistance mechanism during the treatment. To our knowledge, this is the first report of IS*Kpn26* element mediating *mgrB* disruption in the ST1 colistin and CRKP obtained from a patient with chest infection in mainland China. This study provides new research ideas to explore the clinical drug resistance mechanism of CRKP and the critical need to monitor and understand resistance mechanisms to preserve the efficacy of last-line antibiotics such as colistin.

**IMPORTANCE:**

Of note, this chapter gives an update on colistin resistance in sequence type 1 *Klebsiella pneumoniae*, by focusing on the *mgrB* disrupted by IS*Kpn26* element.

## INTRODUCTION

*Klebsiella pneumoniae* is a common Gram-negative opportunistic pathogen involved in various infections, including pneumonia, bacteremia, liver abscesses, and urinary tract infections (1). *K. pneumoniae* is often resistant to several antibacterial agents, including carbapenems, cephalosporins, quinolones, sulfamethoxazole/trimethoprim, and aminoglycosides. The widespread carbapenem-resistant *Klebsiella pneumoniae* (CRKP) isolates are the leading cause of antimicrobial resistance, which is a critical threat to public health. For CRKP, cefepime, ceftazidime, cefotaxime, imipenem, meropenem, and ertapenem showed high resistance rates of 96.3%, 99.0%, 99.8%, 97.2%, 97.8%, and 99.7%, respectively (2, 3). However, CRKP, colistin, ceftazidime–avibactam, and tigecycline showed high susceptibility rates of 89.9%, 90.2%, and 96.5%, respectively. Colistin is an antibiotic of the polymyxin family that has experienced a resurgence in the last decade for the treatment of multidrug-resistant Gram-negative bacteria. Colistin is considered a last-line antibiotic for the treatment of CRKP infections. However, the cases of hypervirulent colistin-resistant CRKP are increasing in clinical practice in China (4–6). Colistin is a cationic antimicrobial peptide that binds to the lipid A phosphate moiety of bacterial lipopolysaccharide, resulting in the leakage of intracellular components from the cell membrane.

Common mechanisms mediating colistin resistance in *K. pneumoniae* include chromosomal mutations of the genes associated with the modification of lipid A, such as those encoding the enzymes involved in lipid A synthesis and the PmrAB and PhoPQ two-component systems (7). Other resistance strategies include the efflux pump mechanism (AcrAB–TolC and SoxSR), which is similar to many of the tigecycline resistance mechanisms described (7). For m*cr-1*-positive isolates, amikacin and trimethoprim/sulfamethoxazole showed limited activity, with susceptibility rates of 18.7% and 23.1%, respectively. *mgrB* is also an important mechanism of colistin resistance in *K. pneumoniae*, often associated with *mgrB* mutations (8). Recently, disruption of the *mgrB* gene by insertion sequences (ISs) such as IS*Kpn25*, IS*Kpn26*, IS*Kpn14*, and IS*903B* was reported to be an important mechanism mediating colistin resistance in *K. pneumoniae* (9, 10). Significantly, colistin resistance mediated by disruption of the *mgrB* gene has not been reported in ST1 *K. pneumoniae*; most of the disruption of the *mgrB* in *K. pneumoniae* was associated with ST11 and ST152 (6, 11, 12). This study reports five CRKP isolates isolated from the sputum of an infected patient with acute cerebral infarction. ST clones, resistance genes, and genetic characteristics of five *K. pneumoniae* isolates were analyzed by whole genome sequencing and bioinformatics techniques. This is the first report of the IS*Kpn26* element mediating *mgrB* disruption in the ST1 colistin and CRKP recovered from a patient with a chest infection in mainland China. This study provides new research ideas to explore the clinical drug resistance mechanism of CRKP.

## MATERIALS AND METHODS

### Case information

**Fig 1:**
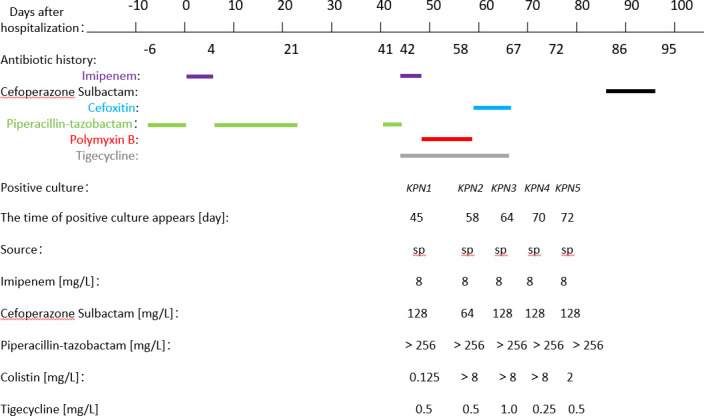
Antibiotic history and the results of microbiology cultures. (The doses of antibiotics are as follows: piperacillin–tazobactam, 4.5 g every 8 h, 6 days before admission to day 1; imipenem, 1 g every 8 h, day 1 to day 4; piperacillin–tazobactam, 4.5 g every 8 h, day 5 to day 21; no antibiotics, day 22 to day 40; piperacillin–tazobactam, 4.5 g every 8 h, day 41 to day 42; imipenem, 1 g every 8 h, day 42 to day 44; tigecycline, 50 mg every 12 h, day 42 to day 44; polymyxin B, 0.5 million units every 12 h, day 45 to day 58; tigecycline, 50 mg every 12 h, day 45 to day 58; cefoxitin, 2 g every 8 h, day 59 to day 67; tigecycline, 50 mg every 12 h, day 59 to day 67.) KPN1, A2303300070, isolated on day 45; KPN2, A2304130040, isolated on day 58; KPN3, A2304210063, isolated on day 64; KPN4, A2304240052, isolated on day 70; KPN5, A2304290062, isolated on day 72; sp, sputum.

A 72-year-old man was admitted to the ICU of a local hospital for acute cerebral infarction. Prior to this admission, he received anti-infective treatment with piperacillin–tazobactam (4.5 g, q8h) for 6 days before this admission. After this admission, the anti-infective regimen was switched to imipenem cilastatin sodium (1 g, q8h, ivgtt) for 4 days until the lung exudation improved. The patient continued anti-infective treatment with piperacillin–tazobactam (4.5 g, q8h) for another 17 days. After 3 weeks, the patient’s symptoms of infection improved compared with before, and the patient was transferred to the rehabilitation ward. During the first 10 days in the rehabilitation ward, no antimicrobial agents were used. However, the patient developed a severe infection and pneumonia on day 41, he had received anti-infective treatment with piperacillin–tazobactam (4.5 g, q8h) for 2 days, the patient was admitted to the ICU, and the anti-infective regimen was switched to imipenem cilastatin sodium (1 g, q8h, ivgtt) combination with tigecycline (50 mg, q12h, ivgtt) for 2 days. On day 45, KPN1 (CRKP) was isolated from sputum culture. The anti-infective regimen was switched to polymyxin B sulfate (500,000 IU, q12h, ivgtt) combination with tigecycline (50 mg, q12h, ivgtt) for 13 days, based on the results of antimicrobial susceptibility testing. However, after 14 days of treatment with polymyxin B sulfate and tigecycline, KPN2, a colistin-resistant CRKP [minimum inhibitory concentration (MIC) >8 mg/L] was isolated from a subsequent sputum sample, and the anti-infective regimen was changed to tigecycline (50 mg, q12h, ivgtt) and cefoxitin sodium (2 g, q6h, ivgtt) for 9 days. During the treatment period, on day 64, KPN3, the carbapenem-resistant *K. pneumoniae* isolates isolated from sputum samples were resistant to colistin. On day 70, KPN4, the carbapenem-resistant *K. pneumoniae* isolates isolated from sputum samples were still resistant to colistin. On day 72, after the discontinuation of polymyxin B sulfate 14 days later, the sensitivity of *K. pneumoniae* (KPN5) to colistin recovered, with the MIC decreasing to 2 mg/L (MIC = 2 mg/L) (Fig. 1).

### Clinical isolates

The five non-duplicate *K. pneumoniae* isolates were isolated from a 72-year-old male patient who was admitted to the Second Affiliated Hospital of Jiaxing University in Jiaxing, China, from 30 March 2023 to 29 April 2023. Samples were collected from the patient’s lower respiratory tract at different points during the colistin treatment, which were tested using routine aerobic culture techniques in the Department of Laboratory Medicine, the Second Affiliated Hospital of Jiaxing University. The *K. pneumoniae* isolate A2303300070 was isolated before the use of colistin, while the remaining four *K. pneumoniae* isolates were isolated after colistin treatment (Table 1). All bacterial isolates were routinely cultured on Columbia agar containing 5% sheep blood and incubated at 35°C–37°C for 18–24 h. Individual *K. pneumoniae* isolates were routinely cultured; species identification was confirmed by the matrix-assisted laser desorption/ionization–time-of-flight mass spectrometry system (bioMérieux, France), using the *K. pneumoniae* isolates growing on Columbia agar. The process can be summarized as follows: (i) A *K. pneumoniae* colony is transferred to the sample plate, which will later be exposed to laser irradiation. (ii) The plate is then irradiated using the laser, and the proteins in the bacteria are ionized. (iii) The time required for these ionized proteins to fly to the detector determines the mass-to-charge ratio (m/z) of the component proteins, and the intensity of the signal provides the mass spectrum. (iv) The mass spectrum obtained is then compared with those in a reference database to identify the bacteria.

**TABLE 1:** Susceptibility of five CRKP clinical isolates to antimicrobial agents^*a*^

Number	Bacterial isolate	MIC (mg/L)														
		IPM	MEM	FEP	CAZ	CRO	ATM	CIP	SCF	CZA	COL	TGC	AMK	SXT	FOS	TZP
KPN1	A2303300070	8	16	＞128	＞32	＞32	＞128	＞8	128	0.5	0.125	0.5	1	4	>128	＞256
KPN 2	A2304130040	8	16	64	＞32	＞32	＞128	＞8	64	0.25	＞8	0.5	1	2	>128	＞256
KPN 3	A2304210063	8	16	＞128	＞32	＞32	＞128	＞8	128	1	＞8	1	1	8	>128	＞256
KPN 4	A2304240052	8	64	＞128	＞32	＞32	＞128	＞8	128	0.5	＞8	0.25	1	8	>128	＞256
KPN 5	A2304290062	8	16	＞128	＞32	＞32	＞128	＞8	128	1	2	0.5	1	4	>128	＞256

^*a*^ IPM, imipenem; MEM, meropenem; FEP, cefepime; CAZ, ceftazidime; CRO, ceftriaxone; ATM, aztreonam; CIP, ciprofloxacin; SCF, cefoperazone/sulbactam; CZA, ceftazidime–avibactam; COL, colistin; TGC, tigecycline; AMK, amikacin; SXT, sulfamethoxazole; FOS, fosfomycin; TZP, piperacillin–tazobactam.

### Antimicrobial susceptibility testing

The MICs of 15 antibacterial drugs were tested by the broth microdilution method using Sensititre Gram-negative panels (Thermo Scientific, Co., Ltd., Shanghai, China). The ranges for antibacterial agents tested were as follows: imipenem (0.06–128 mg/L), meropenem (0.03–64 mg/L), ceftazidime–avibactam (0.03–64 mg/L), cefepime (0.06–128 mg/L), tigecycline (0.06–32 mg/L), amikacin (1–128 mg/L), ceftazidime (0.25–32 mg/L), ceftriaxone (0.25–32 mg/L), aztreonam (1–128 mg/L), ciprofloxacin (0.06–8 mg/L), cefoperazone/sulbactam (1–128 mg/L), piperacillin–tazobactam (2–256 mg/L), colistin (0.125–8 mg/L), sulfamethoxazole (0.25–16 mg/L), and fosfomycin (1–1,024 mg/L). The MIC was defined as the lowest concentration of an antimicrobial agent that will inhibit the visible growth of a microorganism after overnight incubation. Testing was done as follows. (i) The bacterial isolates were routinely cultured on Columbia agar containing 5% sheep blood and incubated at 35°C–37°C for 18–24 h. (ii) Cation-adjusted Mueller–Hinton II broth (CAMHB; BD) was used in this study. (iii) The 0.5 McFarland (Mc) concentration of the bacterial suspension was inoculated in CAMHB with final cell turbidity of 5 × 10^5^ CFU/mL in panels. (iv) Ambient incubation of isolates in panels was performed at 35°C–37°C for 16–18 h. (v) The results were interpreted according to the Clinical and Laboratory Standards Institute (CLSI-M100) guidelines for all antimicrobials except for tigecycline, which was interpreted using U.S. Food and Drug Administration MIC breakpoints. *Escherichia coli* ATCC 25922 was used as a quality control isolate.

### String tests

The experimental isolates were inoculated onto Columbia agar containing 5% sheep blood and incubated at 35°C for 18–24 h. The inoculation ring gently touched the surface of a single colony and then pulled upward. The string test is considered positive, indicating a hyperviscosity phenotype if a viscous string measuring >5 mm in length is obtained by stretching bacterial colonies on an agar plate as previously described (13, 14).

### Short-read genome sequencing and analysis

Genomic DNA was extracted using the Tianamp Bacteria DNA Kit [Tiangen biochemical technology (Beijing) Co., Ltd.] and subjected to Illumina paired-end sequencing (Illumina Inc., San Diego, CA). Raw reads were trimmed using fastp v0.23.2. The trimmed reads were assembled using Unicycler v0.5.0 with default settings. Gene predictions and functional annotations were performed using Prokka v1.14.5. Sequence typing, capsule typing, and screening for virulence factors and acquired antimicrobial resistance genes were performed using Kleborate v.2.0.1. MOB-suite v.3.0.1 was used to predict plasmid sequences from the hybrid assemblies and identify their respective replicon types. The presence of resistance genes was detected using both CARD and ResFinder databases. Virulence factors were predicted using Abricate based on the Virulence factors of pathogenic bacteria (VFDB) database.

## RESULTS

### Antimicrobial susceptibility testing

All five isolates (A2303300070, A2304130040, A2304210063, A2304240052, and A2304290062) showed comparable patterns of drug resistance (Table 1). The MIC values for the five isolates of imipenem MIC were 8 mg/L; the MIC values for meropenem were 16L, 16, 16, 64, and 16 mg/L; the MIC values for cefepime were >128, 64, >128, >128, and >128 mg/L, respectively. The MIC values for colistin were 0.125, >8, >8, >8, and 2 mg/L, respectively. Of note, isolate A2303300070 and isolate A2304290062 showed susceptibility to colistin, and the remainder showed resistance to colistin.

### Antimicrobial resistance, virulence factors, gene profiles, and MLST typing

The isolates were all positive for the string test. Whole genome sequencing of the virulence factors was compared with the VFDB (virulence factors of pathogenic bacteria, a reference database for bacterial virulence factors, doi:10.1093/nar/gki008), which indicated that the isolates were hypervirulent with the virulence genes *ybtA*/*E*/*Q*/*S*/*T*/*U*/*X* encoding yersiniabactin and virulence genes *entA/B/C/D/E/F/S* encoding enterotoxin. All five CRKP isolates carried the carbapenemase gene *bla*_KPC-2_ and *bla*_CTX-M-55_/*bla*_SHV-1_/*bla*_TEM-104_ encoding β-lactamases. In addition, all five isolates harbored *fosA*, *oqxA*/*oqxB*, *tet-34*, the aminoglycoside antibiotic resistance gene *AAC(3′)-IId,* and *dfrA14*. Single-nucleotide polymorphism (SNP) revealed that all five *K*. *pneumoniae* isolates differed by less than 50 SNP_S_, and the susceptibility of the bacteria to drug susceptibility changed during treatment, suggesting that the five *K*. *pneumoniae* isolates belong to the same clone. By combining the seven housekeeping genes, all five isolates sharing the same sequence type (ST) belonged to ST1.

### Genetic surroundings of *bla*_KPC-2_ and *mgrB*

The *bla*_KPC-2_ was identified within the Tn1721 transposon unit, flanked by the ISE*kpn6* upstream and IS9*kpn8* downstream transposable elements, and through further comparison, we found that all *bla*_KPC-2_ in this study were located in Tn*1721*-mediated A2-type transposons, which is a common transposon type of *bla*_KPC-2_ in China. It was co-mediated by Tn*1721* transposon and IS*26*. Analysis of the gene structure of *mgrB* showed that the gene structure of *mgrB* was not disrupted in two colistin-sensitive *K. pneumoniae* isolates, but the *mgrB* gene in the other three CRKP isolates was truncated by ISkpn26.

## DISCUSSION

According to the CHINET surveillance network, the resistance rate of *K. pneumoniae* to meropenem increased from 2.9% in 2005 to 27.1% in 2021, and that to imipenem increased from 3.0% to 25.5% (15). In Europe, CRKP isolates are most common in the Mediterranean and Balkan countries, with prevalence rates of 60% in Greece and 40% in Italy (16). CRKP is one of the seven multidrug-resistant bacteria in the world, with more than 50,000 deaths due to drug resistance (17). It showed that male sex, ICU admission, lung disease (primary or secondary diagnosis of lung disease recorded in the medical record), and infection with isolates carrying the *bla*_KPC_ gene were risk factors for CRKP-infected patients (18–20). The clinical case in this study was a male patient in ICU who was treated with polymyxin B sulfate 500,000 IU, Q12H in combination with tigecycline 50 mg, Q12H for the carbapenem-resistant *K. pneumoniae* producing *bla*_KPC-2_. Colistin, tigecycline, and ceftazidime–avibactam are important treatment options for patients infected with multidrug-resistant and pan-drug-resistant Gram-negative bacteria, especially for patients with severe CRKP infection (21).

**Fig 2:**
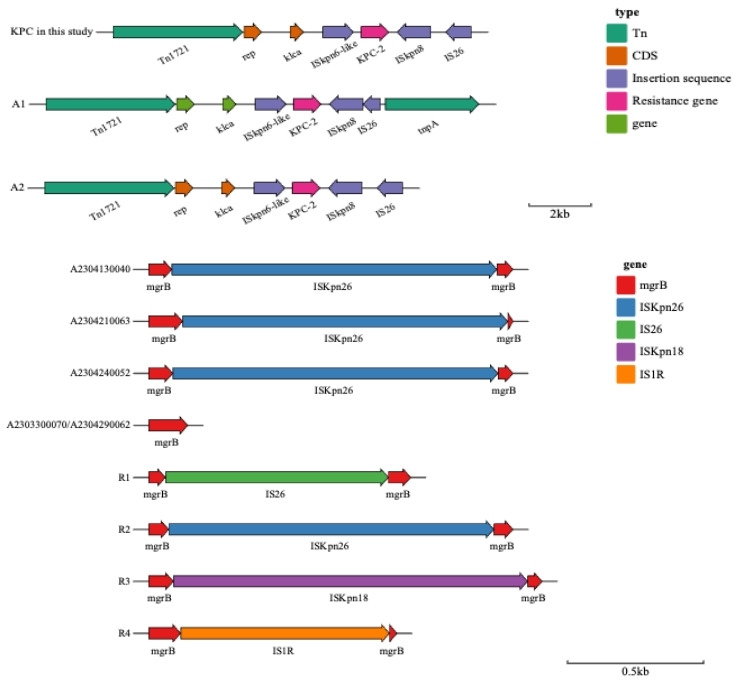
Genetic environment of *bla*_KPC-2_ and *mgrB* in five CRKP isolates and comparative analysis. (**A**) The upstream and downstream of *bla*_KPC-2_ in this study and the genetic characteristics of Tn1721-mediated transposons (A1/A2) in China. (**B**) The *mgrB* structure of isolates A2304130040/A2304210063/A2304240052, which were all resistant to colistin, was disrupted by IS*kpn26* element (1,196 bp), whereas the *mgrB* structure of isolates A2303300070/A2304290062, which were all sensitive to colistin, was complete. R1~R4 represent common genetic environments of *mgrB* in CRKP.

Microbes have evolved unique physiology and genetics to interact dynamically with extreme environments, such as in antibiotic selection conditions, for their adaptation and survival. Drug resistance was one of several adaptive features of microbes in stressed conditions. CRKP resistance to colistin is low but increasing. Between 2002 and 2013, colistin resistance increased from 3.6% to 9.7% in Tunisia and from 1.1% to 2.2% in Europe (22, 23). However, in Dubai and Italy, the colistin resistance rate in CRKP reached 27% and 43%, respectively (24). Available studies (25, 26) suggest that there are two primary mechanisms of bacterial resistance to colistin: plasmid-mediated *mcr* genes and intrinsic resistance mechanisms, such as mutations in regulatory genes such as *PhoPQ/PmrAB*, mainly in the *mgrB* gene. The *mgrB* has been implicated in the feedback regulation of PhoPQ, and point mutations in the *mgrB* gene promote the development of colistin resistance (27). In this study, five carbapenem-resistant *K. pneumoniae* isolates isolated from an infected patient were reported. It was also found that *K. pneumoniae* evolved from colistin-susceptible (MIC = 0.125 mg/L) to resistant (MIC >8 mg/L) within 2 weeks of treatment. However, *K. pneumoniae* regained susceptibility to colistin (MIC = 2 mg/L) 16 days after the end of colistin treatment. The results of second-generation sequencing indicated that the colistin-resistant isolates had a disrupted *mgrB* gene due to the insertion of IS*Kpn26*, in contrast to the colistin-sensitive isolates. According to the literature (4, 28), I*S26/*IS*Kpn18/*IS*1R/*IS*Kpn26* were frequently associated with *mgrB* gene disruption (R1~R4 showed the common genetic environment of *mgrB* in CRKP in Fig. 2B). In China, the primary mechanism of colistin resistance in CRKP is the disruption of the *mgrB* gene by insertion sequences, with IS*Kpn26* being the most common type of insertion sequence (29).

Multilocus sequence typing (MLST) typing analysis identified ST1 among five isolates. A review of the literature showed that the most common STs were ST11, ST25, ST76, and ST37 in CRKP from pediatric patients in China (30); the most common ST type was ST11 from the ICU in *bla*_KPC-2_ producing CRKP, China (31). ST11 and ST258 were dominant in China and the United States, respectively (32). This study reports the rarity of *bla*_KPC-2_-producing ST1 type *K. pneumoniae*. Only one case of highly virulent *bla*_KPC-2_-producing ST1-*K. pneumoniae* has been reported in China (33). The *bla*_KPC-2_-producing ST1 type clones are the first to be found among colistin-resistant *K. pneumoniae*. In China, colistin-resistant ST1-*K. pneumoniae* isolates were isolated from animals as recently as 2007 (34, 35). A cluster of colistin- and carbapenem-resistant ST-*K. pneumoniae* carrying *bla*_NDM-1_ and *mcr-*8.2 was reported in West China Hospital. Colistin- and carbapenem-susceptible ST1-*K. pneumoniae* isolates have also been reported in the Philippines, China, and South Africa (35–38). In contrast, ST1-CRKP isolates (colistin was not determined) were isolated in isolates from Pakistan (39). These ST1-*K. pneumoniae* isolates had previously been described in a clinical *K. pneumoniae* isolate from both animals and humans, suggesting that *K. pneumoniae* clones may spread via the food chain and occupational contact. To our knowledge, there have been no reports of colistin-resistant ST1-*K. pneumoniae* resulting from the disruption of the *mgrB* gene, either in this country or abroad.

CRKP is a major global public health challenge due to its high prevalence and spread. The bacterium causes infections for which there are very few antimicrobial drugs available, resulting in a high morbidity and mortality rate among infected patients. The development of new antimicrobial agents to treat infections caused by drug-resistant bacteria is important; it is also critical to optimize the use of existing antimicrobial agents to enhance their antimicrobial activity. To cope with bacterial mutations during treatment and prevent treatment failure, future research should focus on antimicrobial dose adjustment, bacterial resistance monitoring, and the development of multidrug anti-infective treatment protocols.

### Conclusion

This study reports on five CRKP isolates isolated from the sputum of an infected patient with acute cerebral infarction. ST clones, resistance genes, and genetic characteristics of five *K. pneumoniae* isolates were analyzed by whole genome sequencing and bioinformatics techniques. This is the first report of the IS*Kpn26* element mediating *mgrB* disruption in the ST1 colistin and CRKP recovered from a patient with a chest infection in mainland China. We reported for the first time the co-existence of *bla*_KPC-2_, *mgrB* disruption in a cluster of ST1-*K. pneumoniae* isolates. This study provides new research ideas to explore the clinical drug resistance mechanism of CRKP.
